# Pregnancy-induced gene expression changes in vivo among women with rheumatoid arthritis: a pilot study

**DOI:** 10.1186/s13075-017-1312-2

**Published:** 2017-05-25

**Authors:** Dana E. Goin, Mette Kiel Smed, Lior Pachter, Elizabeth Purdom, J. Lee Nelson, Hanne Kjærgaard, Jørn Olsen, Merete Lund Hetland, Vibeke Zoffmann, Bent Ottesen, Damini Jawaheer

**Affiliations:** 1 UCSF Benioff Children’s Hospital Oakland, Children’s Hospital Oakland Research Institute, 5700 Martin Luther King Jr. Way, Oakland, CA USA; 2 University of California, Berkeley, Berkeley, CA USA; 3 Juliane Marie Center, Copenhagen University Hospital, Rigshospitalet, Copenhagen, Denmark; 4 California Institute of Technology, Pasadena, CA USA; 5 Fred Hutchinson Cancer Research Center, Seattle, WA USA; 6 University of Washington, Seattle, WA USA; 7 University of California, Los Angeles, Los Angeles, CA USA; 8 Aarhus University, Aarhus, Denmark; 9 DANBIO Registry and Copenhagen Centre for Arthritis Research, Centre for Rheumatology and Spine Diseases (VRR), Rigshospitalet, Glostrup, Denmark; 10 Faculty of Health and Medical Sciences, University of Copenhagen, Copenhagen, Denmark; 11 University of California, San Francisco, San Francisco, CA USA

**Keywords:** Rheumatoid arthritis, Pregnancy, RNA-seq, Gene expression, Type I interferon

## Abstract

**Background:**

Little is known about gene expression changes induced by pregnancy in women with rheumatoid arthritis (RA) and healthy women because the few studies previously conducted did not have pre-pregnancy samples available as baseline. We have established a cohort of women with RA and healthy women followed prospectively from a pre-pregnancy baseline. In this study, we tested the hypothesis that pregnancy-induced changes in gene expression among women with RA who improve during pregnancy (pregDAS_improved_) overlap substantially with changes observed among healthy women and differ from changes observed among women with RA who worsen during pregnancy (pregDAS_worse_).

**Methods:**

Global gene expression profiles were generated by RNA sequencing (RNA-seq) from 11 women with RA and 5 healthy women before pregnancy (T0) and at the third trimester (T3). Among the women with RA, eight showed an improvement in disease activity by T3, whereas three worsened. Differential expression analysis was used to identify genes demonstrating significant changes in expression *within* each of the RA and healthy groups (T3 vs T0), as well as *between* the groups at each time point. Gene set enrichment was assessed in terms of Gene Ontology processes and protein networks.

**Results:**

A total of 1296 genes were differentially expressed between T3 and T0 among the 8 pregDAS_improved_ women, with 161 genes showing at least two-fold change (FC) in expression by T3. The majority (108 of 161 genes) were also differentially expressed among healthy women (*q*<0.05, FC≥2). Additionally, a small cluster of genes demonstrated contrasting changes in expression between the pregDAS_improved_ and pregDAS_worse_ groups, all of which were inducible by type I interferon (IFN). These IFN-inducible genes were over-expressed at T3 compared to the T0 baseline among the pregDAS_improved_ women.

**Conclusions:**

In our pilot RNA-seq dataset, increased pregnancy-induced expression of type I IFN-inducible genes was observed among women with RA who improved during pregnancy, but not among women who worsened. These findings warrant further investigation into expression of these genes in RA pregnancy and their potential role in modulation of disease activity. These results are nevertheless preliminary and should be interpreted with caution until replicated in a larger sample.

**Electronic supplementary material:**

The online version of this article (doi:10.1186/s13075-017-1312-2) contains supplementary material, which is available to authorized users.

## Background

Rheumatoid arthritis (RA) is a systemic inflammatory disease characterized by inflammation of the joints that continues to contribute significantly to the global burden of disease [1]. To date, its etiopathogenesis remains unknown, and despite major advancements in treatment, a cure remains elusive. Intriguingly, however, it is well documented that pregnancy can induce significant changes in RA disease activity. During pregnancy, 50–75% of women with RA experience a natural and often dramatic improvement in disease, whereas others may worsen or show no change [2–5]. Some pregnancy-related factors, including maternal-fetal disparity at the human leukocyte antigen locus [6] and fetal microchimerism [7, 8], have been associated with the pregnancy-induced improvement of RA symptoms. Nonetheless, the biology of human pregnancy in terms of the overall systemic changes that it induces, how those may differ between RA and healthy women, and how the pregnancy-induced changes may have an impact on RA remain unknown.

Gene expression studies, more specifically comparison of gene expression profiles (third trimester vs pre-pregnancy baseline) from women followed longitudinally from the pre-pregnancy state to the third trimester, can provide information about biological changes that are induced during pregnancy. Unfortunately, there have been no gene expression studies in which women — healthy or with RA — were studied before they became pregnant as well as during pregnancy to examine such pregnancy-induced changes. Only few studies of gene expression have been conducted in the context of RA pregnancy, using microarray technology [9–12]. In these studies, pregnancy-related changes with respect to the pre-pregnancy state were not examined, due to pre-pregnancy samples not being available [9–11], or pregnancy profiles were compared to profiles of unrelated non-pregnant women [12].

We have established a cohort of RA and healthy women enrolled before pregnancy and followed prospectively through pregnancy. Using state-of-the-art RNA sequencing (RNA-seq) technology, we examined systemic global gene expression in peripheral blood in a subset of women from this cohort as a pilot. We previously identified genes whose expression are modulated by pregnancy in both RA and healthy women, and we showed that a few genes are modulated differently in RA compared to healthy women [13]. We have now used differential expression analysis to further investigate pregnancy-induced gene expression patterns and fold-changes (FCs) in expression within our pilot dataset, taking into account whether the women with RA improved or worsened during pregnancy. We hypothesized that pregnancy-induced changes in gene expression among women with RA who improve during pregnancy may overlap substantially with those observed among healthy women and may differ from changes among women with RA who worsen during pregnancy.

## Methods

### Study subjects

Women with RA and healthy women of Danish descent were recruited and enrolled in a pregnancy cohort in Denmark, as previously described [13]. A subset of 20 women with RA and 5 healthy women from this cohort were included in the pilot dataset. Subjects with RA fulfilled the 1987 revised American College of Rheumatology criteria for RA [14]. The study was approved by the ethics committee for Region Hovedstaden (Denmark), the Danish Data Protection Agency, and the Children’s Hospital Oakland Research Institute Institutional Review Board. All subjects provided written informed consent prior to enrollment.

### Data

Data collected before pregnancy (T0) and at the third trimester (T3) were included in the present study. Therefore, women who had missing data at the T0 baseline or at ﻿T3 (eight women with RA) were excluded from further analyses. At both time points, the women with RA were examined by trained study nurses. Disease activity measures including tender and swollen joint counts based on a total of 28 joints (TJC28 and SJC28, respectively) and patient global health (GH) scores were recorded, and a serum sample was collected for measurement of C-reactive protein (CRP) levels. Data on medication use during the 3 months prior to both visits were also recorded. At each visit, blood samples from the women with RA and healthy women were collected in PAXgene RNA tubes (PreAnalytiX, Hombrechtikon, Switzerland).

### Assessment of RA disease activity

RA disease activity was assessed using the Disease Activity Score based on 28 joints and 4 variables﻿ (DAS28-CRP4, abbreviated henceforth as DAS28), as follows [15]:

$$ \mathrm{D}\mathrm{A}\mathrm{S}28 = \left(0.56*\sqrt{TJC28}\right)+\left(0.28*\sqrt{SJC28}\right)+\left(0.36* ln\left( CRP+1\right)\right)+0.014*\mathrm{G}\mathrm{H}+0.96 $$

Change in DAS28 from T0 to T3 was calculated as: ΔDAS28 = DAS28_T3_ − DAS28_T0_.

The women with RA were categorized into two subsets: those with negative ΔDAS28 values were considered to show an improvement in DAS28 score at T3 compared to the T0 baseline (referred to as the pregDAS_improved_ subset), whereas those with positive ΔDAS28 values were included in the “worsened” subset, referred to as pregDAS_worse_. Any improvement in DAS28 scores (ΔDAS28 < 0) was used as a surrogate for improvement in disease activity. This was done to capture even small changes in disease activity that may correlate with biological changes observed at the gene expression level. Disease activity categories (remission, low, moderate, or high) were assessed using previously defined criteria [16].

### Sample processing and bioinformatic analyses

Total RNA was isolated from frozen blood samples, and barcoded ﻿cDNA libraries were prepared as previously described [13]. Pseudo-alignment of the de-multiplexed raw sequence reads (FASTQ format) to the Ensembl reference human GRCh38 transcriptome assembly, and quantification of transcript abundances was performed using kallisto (version 0.42.4) [17]. BioMart annotations were used to combine transcript-level counts into gene-level estimates. Pseudogenes, genes with no annotations, and genes with very low read counts (<1 count per million) in at least 25% of all samples were filtered out. Any globin genes and ribosomal RNA (rRNA)﻿ transcripts still present were also filtered out. To adjust for variable sequencing depths across samples, the gene-level counts were normalized using the Trimmed Mean of M values (TMM) algorithm as implemented in the edgeR package (version 3.10.5) [18, 19]. To assess batch effects, normalized gene counts from each pair of technical replicates were plotted, and correlations were assessed.

### Statistical analyses

Differential gene expression analysis was performed using edgeR (version 3.10.5) [18] to compare normalized gene-level counts between the T3 and T0 time points *within *each group of women (i.e., pregDAS_improved_, pregDAS_worse_ and healthy women). A model with a paired-sample design was used for these *within*-group comparisons. *Between*-group comparisons (pregDAS_improved_ vs pregDAS_worse_, and pregDAS_improved_ vs healthy) at each time point were also performed using differential expression analyses. A negative binomial distribution was used to handle the over-dispersion in RNA-seq gene counts. Differential expression was tested using generalized linear model (GLM) likelihood ratio tests. To correct for any batch effects, batch was included as a covariate in the model. A *q* value threshold of 0.05 was used to assess significance. Because sample sizes were small, FCs in expression were also used in the interpretation of results, focusing on genes with at least a two-fold change in expression from T0 to T3.

### Functional analysis

Differentially expressed genes were analyzed for over-representation of Gene Ontology (GO) categories using a hypergeometric test implemented in the Web-based Gene Set Analysis Toolkit (WebGestalt) with a threshold of at least five genes per category [20]. A significance threshold of q<0.05 was used to define enrichment. Functional enrichment of gene sets was examined using the STRING database of known and predicted interactions among proteins [21, 22].

## Results

### Study subjects

**Fig. 1:**
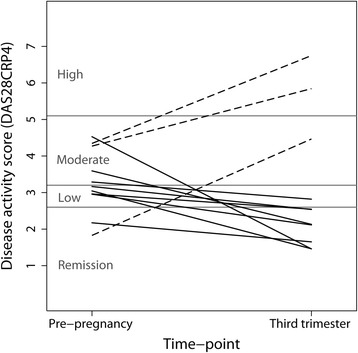
Disease activity before pregnancy and at the third trimester among the women with rheumatoid arthritis. Disease Activity Scores based on 28 joints and 4 variables (DAS28-CRP4) are shown for the eight women who improved during pregnancy (solid lines) and the three women who worsened (dashed lines), at the pre-pregnancy baseline and at the third trimester. (The straight lines between time-points are included for graphical purposes only and do not imply a linear change in score.)

Of the 12 women with RA who had data at both T0 and T3, 8 were in the pregDAS_improved_ group and 3 were in the pregDAS_worse_ group. One woman was excluded because, although she had an increase in DAS28 at T3, she was in remission at both time points and hence did not fit into the pregDAS_worse_ group. The DAS28 scores of the 11 women included in the analyses are shown in Fig. 1. The average disease duration among the women with RA was (mean ± SD) 5.9±4.4 years for pregDAS_improved_ and 8.7±1.0 years for pregDAS_worse_. The average age at conception was 30.3±5.7 years for pregDAS_improved_, 33.2±1.9 years for pregDAS_worse_, and 31.2±5.7 years for the healthy women.

Among the eight pregDAS_improved_ women, three did not take any medications at T0 and only one of these three women started taking medications by T3 (prednisolone + sulfasalazine). The remaining five women were taking prednisolone and/or sulfasalazine at both time points; one of them was also on anti-tumor necrosis factor (anti-TNF) therapy at T0, and another was taking methotrexate at T0. The three pregDAS_worse_ women were all on anti-TNF therapy and taking prednisolone and/or sulfasalazine (except for one) at T0; at T3, one was taking prednisolone + sulfasalazine, one remained on anti-TNF therapy, and one stopped taking medications.

### Genes differentially expressed between T3 and T0 in the pregDAS_improved_ subset

A total of 1296 genes showed significant differential expression between T3 and T0 in the pregDAS_improved_ subset, of which 161 displayed two-fold or more change in expression (*see* Additional file 1). These 161 genes were enriched in a number of immune-related pathways, as shown in Table 1. Similar results were obtained when the woman who was receiving anti-TNF therapy at T0 was excluded from the analysis.

**Table 1:** Gene Ontology (GO) biological processes enriched in genes differentially expressed among women with RA who improved during pregnancy

GO biological process	Gene count	*q* value
Immune system process	55	7.9 × 10^−14^
Response to stress	54	9.1 × 10^−6^
Defense response	35	1.5 × 10^−8^
Immune response	34	2.2 × 10^−8^
Multiorganism process	34	6.7 × 10^−6^
Response to other organism	27	1.4 × 10^−9^
Response to biotic stimulus	27	2.9 × 10^−9^
Innate immune response	22	5.6 × 10^−7^
Response to bacterium	16	9.0 × 10^−6^
Erythrocyte differentiation	10	6.5 × 10^−7^

Of the 161 genes differentially expressed (with FC≥2) between T0 and T3 among pregDAS_improved_ women, several were enriched in GO biological processes relating to immune functions. (*Note*: These 161 genes included some that were also differentially expressed among healthy women and ﻿﻿pregDAS_worse_ ﻿women.)

### Overlap with genes differentially expressed among healthy women

A large proportion of the 161 genes that exhibited at least two-fold change in expression between T3 and T0 among the pregDAS_improved_ women were also differentially expressed among healthy women (*q*<0.05), mostly (*n*=108) with two-fold or more change in expression (*see* Additional files 1 and 2), although some (*n*=33) showed more modest FCs in expression (1.5≤FC<2.0). In addition, another 77 genes differentially expressed (*q*<0.05) in both the pregDAS_improved_ and healthy groups showed lower FCs among the pregDAS_improved_ women (1.5≤FC<2.0) than among the healthy women (FC≥2). The genes with FC≥2 in both the women with RA and healthy women were enriched in immune-related pathways such as immune system process (*q* =7.9×10^−9^), response to other organism (*q* =2.7×10^−7^), and defense response to bacterium (*q* =9.3×10^−6^).

### Overlap with genes differentially expressed in the pregDAS_worse_ subset

Of the 161 genes differentially expressed in the pregDAS_improved_ subset, some (*n*=31) were also differentially expressed (*q*<0.05, FC≥2) among pregDAS_worse_ women, with most of these genes overlapping with those differentially expressed among healthy women (*n*=30) (*see* Additional file 2). The FCs in expression from T0 to T3 were correlated between the two RA subsets (Pearson correlation coefficient *r*=0.86, *p*<0.00005).

### Differences in genes differentially expressed (T3 vs T0) between the pregDAS_improved_ and pregDAS_worse_ subsets

**Fig. 2:**
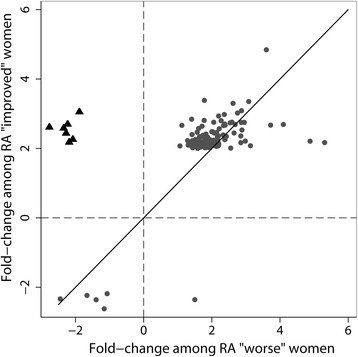
Fold-changes in expression for genes differentially expressed (third trimester [T3] vs baseline [T0]) among women who improved, but not among those who worsened. Fold-changes in expression are plotted for women who improved during pregnancy (*y*-axis) and women who worsened during pregnancy (*x*-axis) only for those genes that were differentially expressed (T3 vs T0) in the “improved” group (*q*<0.05) and not in the “worsened” group. Only genes with FC≥2 are shown. Fold-changes for genes that were under- expressed at T3 are shown as negative values. The majority of genes showed similar expression changes in both groups of women with RA, as shown by the *gray dots* around the *y* = *x* line (*solid line*). A small cluster of genes (*black triangles*), however, was over-expressed among women who improved and under-expressed among women who worsened. Genes in this cluster were *IFI44*, *IFI44L*, *IFIT1*, *HERC5*, *CMPK2*, *RSAD2*, and *SIGLEC1*

**Fig. 3:**
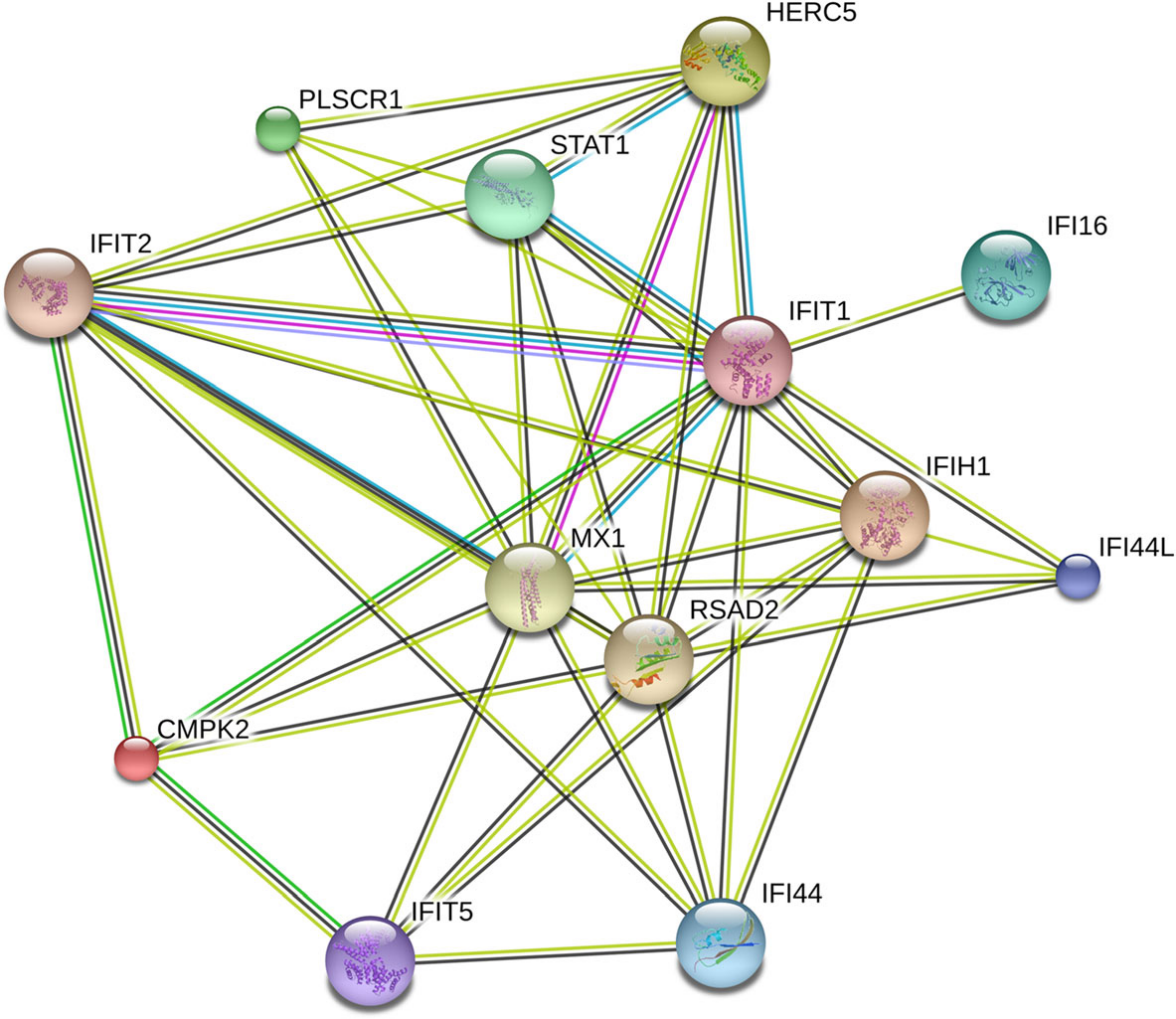
Network graph showing associations between proteins encoded by genes with contrasting changes in expression from the pre﻿-﻿﻿pregnancy baseline to the third trimester between RA women who improved and those who worsened. The nodes in the network represent proteins encoded by genes. The edges represent known as well as predicted interactions between the proteins, suggesting that they are part of a common functional network. Proteins that were not joined in the network are not shown. Variations at the levels of transcripts or post-translational modification are also not shown. Nodes with unknown 3D structure are shown by smaller size

The remaining 130 genes that were differentially expressed (T3 vs T0: FC≥2, *q*<0.05) in our data among the pregDAS_improved_ women, but not among the pregDAS_worse_ women, were significantly enriched in several immune-related GO processes, such as immune system process (*q*=4.7×10^−10^), defense response (*q*=9.6×10^−6^), immune response (*q*=5.0×10^−5^), and innate immune response (*q*=8.0×10^−6^), among others (Table 2). Of these 130 genes, 78 were also differentially expressed among healthy women (*see* Additional files 1 and 2). A comparison of the FCs in expression from T0 to T3 within each of the RA subsets revealed a small cluster of genes that were significantly over-expressed at T3 among pregDAS_improved_ women and under-expressed (FC≥2, not significant) among pregDAS_worse_ women (Fig. 2). By T3, mean normalized expression levels of these genes were higher among the pregDAS_improved_ women than among the pregDAS_worse_ women. Interestingly, all of the genes in this cluster were type I interferon (﻿IFN)-inducible (*IFI44*, *IFI44L*, *IFIT1*, *HERC5*, *CMPK2*, *RSAD2*, and *SIGLEC1*). Among these, only the *RSAD2* gene was differentially expressed (under-expressed) at T3 compared to T0 among healthy women (*q*<0.05, FC≥2). A number of other type I IFN-inducible genes (*STAT1*, *IFI16*, *IFIT2*, *IFIT5*, *IFIH1*, and *MX1*) also exhibited similar patterns of expression, being over-expressed at T3 (*q*<0.05) among pregDAS_improved_ women, though FCs were more modest (1.5- to 1.9-fold), and under-expressed among pregDAS_worse_ women. The *PLSCR1* gene, which can enhance the type I IFN response, also showed a significant increase in expression (FC≥2) in the pregDAS_improved_ women by T3, but no change was observed in the pregDAS_worse_ women. Of interest, a comparison of normalized expression levels of these genes at T0 between pregDAS_improved_ and healthy women revealed that some of the IFN signature genes (*IFI44*, *IFI44L*, *HERC5*, *CMPK2*, *SIGLEC1*, and *MX1*) were significantly under-expressed in the pregDAS_improved_ subset compared to healthy women at T0 (*q*<0.05, FC≥2). However, by T3, there were no significant differences in expression of these genes between the two groups of women. The IFN-inducible genes that were over-expressed at T3 among pregDAS_improved_ women, but not among in the pregDAS_worse_ women, belong to a common functional network, as shown by known and predicted protein interactions from the STRING database (Fig. 3). Other than *RSAD2*, none of the genes shown in this network were differentially expressed (T3 vs T0) among healthy women.

**Table 2:** Gene Ontology biological processes enriched in genes differentially expressed among women with RA who improved during pregnancy, but not among those who worsened

GO biological process	Gene count	*q* value
Immune system process	43	4.7 × 10^−10^
Defense response	26	9.6 × 10^−6^
Immune response	24	5.0 × 10^−5^
Innate immune response	18	8.0 × 10^−6^
Response to other organism	17	6.7 × 10^−5^
Response to biotic stimulus	17	1.0 × 10^−4^
Hemopoiesis	15	2.0 × 10^−4^
Homeostasis of number of cells	11	6.9 × 10^−6^
Myeloid cell differentiation	11	7.1 × 10^−5^
Erythrocyte differentiation	10	2.5 × 10^−7^

The 130 genes that were differentially expressed (with FC≥2) between T0 and T3 among pregDAS_improved_, but not among pregDAS_worse_ women, included several that were enriched in GO biological processes relating to immune functions

## Discussion

There have been no previous reports of pregnancy-induced changes in gene expression in RA or healthy women from pre-pregnancy to the third trimester, other than our own published findings from our pilot RNA-seq dataset [13]. Gene expression changes that accompany improvement or worsening of RA disease activity during pregnancy, relative to a pre-pregnancy baseline, have also not been investigated. Thus, our findings are novel.

In our data, genes showing similar differential expression from T0 to T3 in both the pregDAS_improved_ and pregDAS_worse_ women were also similarly differentially expressed among healthy women, suggesting that these were normal pregnancy-related changes. The observation that a cluster of type I IFN-inducible genes demonstrated contrasting changes in expression from T0 to T3 between the pregDAS_improved_ and pregDAS_worse_ women was particularly interesting. These genes were over-expressed at T3 ﻿﻿among the pregDAS_improved_ women﻿, compared to the T0 baseline, even though at T3 five of the eight pregDAS_improved_ women were on corticosteroid therapy, which has been shown to inhibit type I IFN signaling [23, 24]. Over-expression of IFN-inducible genes has also been observed in RNA-seq profiles of neutrophils among patients with juvenile idiopathic arthritis (JIA) who were in drug-induced remission compared to those with active disease [25]. Neutrophils appear to be activated during pregnancy, as reported by us [13] and others [26–28]. However, neutrophil-related genes were over-expressed in both the pregDAS_improved_ and pregDAS_worse_ groups; yet, levels of the IFN-inducible genes (except *IFI27*) decreased from T0 to T3 in the pregDAS_worse_ subset (not significantly). Thus, over-expression of IFN-inducible genes during pregnancy among pregDAS_improved_ women may not be due to neutrophil activation. Of interest, the three women who worsened during pregnancy were all on anti-TNF therapy at baseline, and non-responders to anti-TNF therapy have been reported to produce lower levels of IFNγ than good responders [29, 30]. Although we might also expect type I IFN genes to be differentially expressed in concert with type I IFN-inducible genes, we found no such evidence in our data.

The significance of the type I IFN signature in the pregDAS_improved_ subset is not entirely clear. Type I IFNs appear to have conflicting roles in autoimmune diseases. Whereas a type I IFN signature in lupus is associated with active disease [31], it correlates with an alleviation of symptoms in multiple sclerosis (MS) [32], and IFNβ therapy has been used successfully to treat patients with MS [33]. In vitro studies as well as studies of animal models of arthritis suggest that type I IFN most likely has a protective role in RA [32, 34], although translation of the findings from the animal models to treat human RA using IFNβ therapy has thus far not been successful [34]. The majority of the IFN-inducible genes identified in our data were under-expressed in the pregDAS_improved_ subset compared to healthy women at T0, which may suggest that expression patterns of these genes in RA could be deregulated as a consequence of the disease. Type I IFNs appear to promote down-regulation of pro-inflammatory cytokines and up-regulation of anti-inflammatory cytokines in organ-targeted autoimmune diseases [32, 35]. It is thus possible that the lower levels of IFN-inducible genes in the pregDAS_improved_ women before pregnancy may reflect lower systemic IFNβ levels, which favor a pro-inflammatory state. Furthermore, while expression levels of the IFN-inducible genes did not change significantly from T0 to T3 among healthy women, they changed significantly in the pregDAS_improved_ women, and by T3, they were no longer differentially expressed between the two groups (data not shown). These observations support the notion that increased levels of the IFN-inducible genes identified in our pilot data may help shift the immunomodulatory balance to an anti-inflammatory state during pregnancy. We speculate that specific levels of these gene products may be crucial to maintaining a healthy pregnancy, explaining why expression levels at T3 among pregDAS_improved_ women became comparable to those of healthy women.

Some of the IFN-inducible genes that were over-expressed at T3 ﻿in our data﻿ (*IFI44*, *IFI44L*, and *SIGLEC1*) have been reported to show increased expression in pregnant RA women compared to unrelated non-pregnant RA women [12]. That study found no association between expression levels of these genes and disease activity. However, pre-pregnancy data on both disease activity and expression levels were not available for comparison with pregnancy data. Although in the present study we did not test for associations between expression of IFN-inducible genes and disease activity, we cannot exclude the possibility that they may be involved.

The strengths of our study include the availability of paired time-dependent data from the same women at both time points. This allowed global gene expression changes induced by pregnancy to be compared to a pre-pregnancy baseline, while at the same time controlling for unmeasured confounders. The use of RNA-seq technology to assess gene expression was also an advantage over microarray data. Our study does have some limitations. First, the sample sizes were small, especially for the women who worsened during pregnancy. However, the availability of data from the same women before and during pregnancy, as well as the ethnic homogeneity of the study population, enabled us to overcome some of the limitations of having a small sample size. Nevertheless, observations relating to the pregDAS_worse_ subset should be interpreted with caution until they can be replicated in a larger sample. Because samples were processed in two batches, we used sample replicates in both batches to assess and mitigate batch effects. We did not examine changes in proportions of different cell types in blood samples across time points, because our goal was to identify overall systemic gene expression changes resulting either from altered expression of specific genes or from differences in cell proportions. Although there is a possibility that anti-TNF and/or other medications may have influenced the results, the lack of variation in medication use within each subset of women with RA precluded us from determining if this was the case. Nevertheless, in the pregDAS_improved_ group, exclusion of the woman on anti-TNF therapy did not significantly change the results, suggesting that the findings were not influenced by anti-TNF. We did not adjust for dosage and/or specific medications.

## Conclusions

The results from our pilot study suggest that genes demonstrating significant changes in expression in the pregDAS_improved_ women, but not the pregDAS_worse_ women, during pregnancy — that is, those showing similar patterns of expression as in healthy pregnancy or those among the IFN signature — could be involved in the natural amelioration of RA. These findings warrant further investigations into expression of these genes in RA pregnancy, but are nevertheless preliminary, and should be interpreted with caution until replicated in a larger sample.

## Additional files

Additional file 1: Table S1. Within-group differential expression results (T3 vs T0) for the 161 genes that showed significant differential expression (*q*<0.05, FC≥2) among the pregDAS_improved_ women. Fold changes (FCs) in expression, *p* values (unadjusted), and *q* values are shown for each of the three groups of women (i.e., pregDAS_improved_, pregDAS_worse_, and healthy women). Given the small sample sizes, we recommend that the *q* values be interpreted with caution, especially among the three pregDAS_worse_ women. (PDF 153 kb)

Additional file 2: Figure S1. Numbers of genes differentially expressed (*q*<0.05, FC≥2) in the three groups of women. (*Note*: These numbers are provided only to give context, given the small sample sizes). (PDF 5 kb)

## Abbreviations

CRP: C-reactive protein
DAS28-CRP4: Disease Activity Score based on 28 joint counts﻿ and 4 variables, including C-reactive protein
FC: Fold-change
GH: Patient global health
GLM: Generalized linear model
GO: Gene Ontology
IFN: Interferon
JIA: Juvenile idiopathic arthritis
MS: Multiple sclerosis
pregDAS_improved_: Women with rheumatoid arthritis whose disease activity improved during pregnancy
pregDAS_worse_: Women with rheumatoid arthritis whose disease activity worsened during pregnancy
RA: Rheumatoid arthritis
RNA-seq: RNA sequencing
rRNA: Ribosomal RNA
SJC28: S﻿wollen joint count based on 28 joints
T0: Pre-pregnancy
T3: Third trimester
TJC28: Tender joint count based on 28 joints
TMM: Trimmed Mean of M values
TNF: Tumor necrosis factor
