# Supplementary material for: Pregnancy-induced gene expression changes in vivo among women with rheumatoid arthritis: a pilot study
Source: Arthritis Res Ther. 2017 May 25;19:104. doi: 10.1186/s13075-017-1312-2 (PMC5445464; doi:10.1186/s13075-017-1312-2)
Supplement: Supplementary file 1 — Within-group differential expression results (T3 vs T0) for the 161 genes that showed significant differential expression (q<0.05, FC≥2) among the pregDASimproved women. Fold changes (FCs) in expression, p values (unadjusted), and q values are shown for each of the three groups of women (i.e., pregDASimproved, pregDASworse, and healthy women). Given the small sample sizes, we recommend that the q values be interpreted with caution, especially among the three pregDASworse women. (PDF 153 kb) [file 13075_2017_1312_MOESM1_ESM.pdf]

Table S1

	RA improved (n=8)			RA worsened (n=3)			Healthy (n=5)		
**Genes**	**FC**	**p-value**	**q-value**	**FC**	**p-value**	**q-value**	**FC**	**p-value**	**q-value**
ABCA13	6.0	1.24E-15	4.21E-13	8.2	3.72E-08	2.72E-05	10.0	1.02E-24	1.41E-21
ABCG1	0.5	1.23E-10	1.54E-08	0.9	7.51E-01	1.00E+00	0.7	1.31E-02	6.58E-02
ACKR1	2.8	4.02E-11	5.57E-09	2.3	6.18E-02	9.47E-01	2.8	4.22E-06	1.09E-04
AIM2	2.1	1.96E-05	7.24E-04	1.5	3.80E-01	1.00E+00	1.7	9.42E-05	1.46E-03
ALAS2	2.7	4.70E-14	1.09E-11	3.7	5.19E-02	9.08E-01	3.7	9.50E-10	7.61E-08
ANK1	2.4	1.57E-11	2.27E-09	2.9	1.96E-02	6.32E-01	3.1	1.41E-13	3.05E-11
ANXA3	2.7	3.55E-06	1.60E-04	4.1	4.28E-03	2.85E-01	5.0	6.76E-28	1.34E-24
AQP1	2.0	2.93E-08	2.27E-06	1.9	7.79E-02	1.00E+00	2.8	1.93E-10	1.90E-08
ARHGEF12	2.4	1.00E-18	7.33E-16	2.1	5.89E-02	9.44E-01	1.8	1.94E-06	5.69E-05
ARHGEF37	2.5	8.27E-09	7.45E-07	2.1	3.21E-02	7.78E-01	2.7	1.82E-06	5.38E-05
ARL4A	2.3	6.84E-16	2.53E-13	1.9	2.68E-02	7.32E-01	2.0	5.73E-05	9.64E-04
ARL6IP1	2.0	2.38E-12	3.79E-10	1.4	2.57E-01	1.00E+00	1.5	6.54E-03	3.98E-02
BCL2L1	2.1	2.69E-11	3.81E-09	1.9	2.18E-01	1.00E+00	2.4	1.13E-08	6.60E-07
BNIP3L	2.2	7.13E-10	7.49E-08	1.4	5.44E-01	1.00E+00	2.0	2.04E-04	2.73E-03
BPGM	3.1	3.02E-25	5.23E-22	2.9	8.99E-03	4.30E-01	3.0	1.22E-11	1.74E-09
BPI	2.5	1.25E-05	4.93E-04	5.1	6.26E-08	4.34E-05	4.8	1.32E-11	1.87E-09
C9orf78	2.4	2.05E-15	6.45E-13	1.7	7.48E-02	1.00E+00	2.7	1.37E-14	3.21E-12
CA1	4.8	0.00E+00	0.00E+00	3.6	6.36E-03	3.61E-01	5.3	5.93E-15	1.52E-12
CAMP	6.5	1.15E-14	2.90E-12	13.7	6.19E-26	8.58E-22	17.8	2.22E-31	1.03E-27
CASP5	2.2	8.37E-04	1.49E-02	1.4	5.78E-01	1.00E+00	1.8	1.05E-05	2.36E-04
CCDC176	2.8	9.58E-33	3.32E-29	2.1	6.41E-03	3.62E-01	2.9	7.82E-12	1.15E-09
CD177	4.2	3.00E-04	6.82E-03	15.4	5.53E-05	1.28E-02	14.2	1.55E-29	5.38E-26
CD24	3.1	3.08E-10	3.50E-08	2.7	7.04E-06	2.50E-03	2.7	2.32E-13	4.73E-11
CDC42BPA	2.1	1.34E-12	2.21E-10	1.6	5.46E-02	9.24E-01	1.8	4.27E-05	7.63E-04
CDK1	2.2	6.94E-07	3.82E-05	2.2	4.89E-03	3.09E-01	1.0	9.43E-01	9.73E-01
CEACAM6	2.5	3.82E-07	2.30E-05	3.9	1.78E-05	5.03E-03	4.7	2.71E-11	3.58E-09
CEACAM8	4.7	3.01E-12	4.69E-10	6.5	5.07E-11	1.00E-07	7.3	3.94E-16	1.33E-13
CEBPE	2.2	5.88E-05	1.84E-03	1.8	1.31E-02	5.09E-01	2.5	2.54E-12	4.24E-10
CETN2	2.3	4.72E-10	5.15E-08	1.9	1.82E-02	6.13E-01	2.3	4.77E-09	3.06E-07
CHIT1	2.3	7.90E-07	4.28E-05	3.4	3.10E-08	2.53E-05	3.4	3.01E-18	1.23E-15
CISD2	2.1	4.46E-19	3.44E-16	1.9	3.43E-02	7.92E-01	1.9	4.22E-05	7.56E-04
CMPK2	2.3	2.69E-04	6.23E-03	0.5	2.92E-01	1.00E+00	0.6	1.77E-02	8.06E-02
CRISP3	7.1	1.43E-21	1.66E-18	12.5	3.14E-19	1.45E-15	18.9	2.74E-28	6.33E-25
CTNNAL1	2.2	5.04E-21	4.99E-18	3.0	2.76E-06	1.16E-03	2.1	1.63E-06	4.96E-05
CTSE	2.5	7.60E-15	1.99E-12	1.6	2.46E-01	1.00E+00	1.8	5.40E-04	5.95E-03
DAAM2	0.4	4.21E-03	4.66E-02	1.5	2.70E-01	1.00E+00	1.5	1.51E-02	7.25E-02
DCAF12	2.2	2.81E-13	5.41E-11	2.0	2.17E-01	1.00E+00	2.0	5.09E-05	8.78E-04
DEFA1	6.8	4.09E-10	4.54E-08	9.7	3.78E-09	4.76E-06	20.8	3.21E-18	1.27E-15
DEFA1B	7.5	2.61E-08	2.04E-06	7.5	7.19E-09	7.12E-06	14.8	1.30E-15	3.85E-13
DEFA3	7.5	1.88E-09	1.91E-07	8.4	1.54E-09	2.13E-06	17.0	9.94E-16	3.07E-13
DPCD	2.1	3.11E-08	2.38E-06	1.1	7.98E-01	1.00E+00	1.7	2.18E-04	2.88E-03
DSC2	2.2	8.62E-05	2.54E-03	2.9	1.15E-02	4.88E-01	3.3	1.87E-22	1.36E-19
DYRK3	2.7	2.87E-22	3.61E-19	2.3	7.63E-03	3.93E-01	2.3	2.40E-06	6.69E-05
E2F2	2.4	2.57E-10	2.95E-08	1.8	1.64E-01	1.00E+00	2.2	9.12E-07	3.07E-05
EPB41	2.3	2.73E-13	5.33E-11	1.8	2.91E-01	1.00E+00	2.1	6.45E-07	2.25E-05
EPB42	2.9	2.68E-20	2.32E-17	2.2	2.70E-02	7.33E-01	3.4	1.13E-15	3.41E-13

	RA improved (n=8)			RA worsened (n=3)			Healthy (n=5)		
**Genes**	**FC**	**p-value**	**q-value**	**FC**	**p-value**	**q-value**	**FC**	**p-value**	**q-value**
FAM104A	2.3	1.13E-18	7.84E-16	1.7	1.16E-01	1.00E+00	2.1	2.22E-07	9.09E-06
FAM210B	2.3	1.74E-17	8.59E-15	2.2	8.26E-02	1.00E+00	2.1	9.68E-06	2.21E-04
FAM46C	3.0	1.66E-13	3.44E-11	2.8	1.32E-01	1.00E+00	2.1	6.36E-05	1.05E-03
FAT1	2.1	1.98E-08	1.62E-06	1.7	7.07E-02	9.87E-01	1.4	2.97E-02	1.15E-01
FCER1A	0.5	1.29E-03	2.02E-02	0.2	8.31E-05	1.77E-02	0.3	4.56E-09	2.94E-07
FCGR1A	2.1	3.82E-04	8.32E-03	2.1	7.81E-02	1.00E+00	1.8	1.21E-06	3.88E-05
FCGR1B	2.1	1.64E-04	4.26E-03	2.1	4.88E-02	8.94E-01	1.9	9.20E-07	3.09E-05
FCGR1C	2.2	9.82E-04	1.66E-02	2.1	5.92E-02	9.45E-01	1.8	2.91E-05	5.51E-04
FECH	2.7	1.09E-12	1.82E-10	2.7	6.15E-02	9.47E-01	2.3	3.79E-07	1.45E-05
FFAR3	2.7	5.12E-04	1.03E-02	1.1	8.43E-01	1.00E+00	2.2	4.48E-06	1.15E-04
FKBP1B	2.1	5.24E-09	4.82E-07	1.4	3.37E-01	1.00E+00	1.8	7.86E-04	7.96E-03
GALNT14	2.4	6.50E-07	3.59E-05	5.2	6.71E-08	4.43E-05	4.1	2.40E-19	1.15E-16
GCLC	2.0	2.83E-15	8.36E-13	1.8	1.51E-02	5.54E-01	1.6	1.22E-04	1.81E-03
GMPR	2.3	1.87E-17	8.81E-15	2.4	1.50E-02	5.54E-01	3.2	3.61E-16	1.25E-13
GPX1	2.3	1.89E-10	2.26E-08	1.6	2.53E-01	1.00E+00	2.5	5.20E-12	8.11E-10
GSPT1	2.2	6.38E-14	1.40E-11	1.3	5.15E-01	1.00E+00	1.9	1.87E-06	5.52E-05
GYPA	2.0	4.12E-13	7.62E-11	1.5	9.85E-02	1.00E+00	2.2	2.54E-05	4.92E-04
GYPB	2.2	7.71E-11	1.01E-08	1.3	3.94E-01	1.00E+00	2.0	3.07E-03	2.24E-02
HEMGN	2.6	2.18E-18	1.44E-15	1.7	2.02E-01	1.00E+00	2.3	4.23E-07	1.59E-05
HEPACAM2	2.3	4.47E-17	1.83E-14	2.4	1.20E-02	4.94E-01	2.6	1.58E-06	4.85E-05
HERC5	2.2	3.60E-03	4.18E-02	0.5	3.19E-01	1.00E+00	0.6	2.59E-02	1.05E-01
HIST3H2BB	2.0	6.80E-16	2.53E-13	1.8	7.88E-03	3.99E-01	2.2	2.35E-08	1.24E-06
HMBS	2.2	2.68E-13	5.31E-11	1.5	1.31E-01	1.00E+00	1.8	1.22E-04	1.81E-03
IFI27	3.4	9.41E-08	6.53E-06	1.8	6.88E-02	9.83E-01	1.6	1.59E-02	7.51E-02
IFI44	2.7	1.66E-04	4.30E-03	0.4	2.22E-01	1.00E+00	0.6	2.86E-02	1.12E-01
IFI44L	2.6	4.61E-04	9.55E-03	0.4	2.69E-01	1.00E+00	0.6	2.59E-02	1.05E-01
IFIT1	2.4	3.18E-03	3.82E-02	0.4	2.92E-01	1.00E+00	0.7	1.76E-01	3.71E-01
IFIT1B	3.2	3.51E-29	9.74E-26	3.1	2.58E-05	6.88E-03	3.0	9.41E-13	1.74E-10
IGHA2	0.4	4.47E-03	4.84E-02	0.7	5.53E-01	1.00E+00	0.6	9.40E-03	5.19E-02
IGHG2	0.4	1.03E-03	1.72E-02	0.6	1.80E-01	1.00E+00	0.7	2.62E-01	4.70E-01
INHBA	3.3	1.03E-09	1.06E-07	3.1	3.52E-04	5.68E-02	3.1	5.16E-07	1.87E-05
ITLN1	4.6	3.22E-34	1.68E-30	4.4	7.55E-06	2.62E-03	5.8	3.14E-29	8.70E-26
KCNH7	2.1	1.46E-04	3.93E-03	3.1	1.17E-02	4.92E-01	3.4	5.90E-16	1.90E-13
KEL	2.2	1.11E-11	1.64E-09	2.0	8.33E-02	1.00E+00	2.4	3.77E-09	2.46E-07
KIF15	2.2	4.76E-09	4.43E-07	1.7	3.03E-02	7.60E-01	1.7	1.44E-02	7.00E-02
KLF1	2.6	1.78E-20	1.65E-17	2.2	4.25E-02	8.50E-01	2.7	6.98E-06	1.69E-04
KRT1	2.2	1.60E-08	1.35E-06	2.0	1.29E-01	1.00E+00	2.8	7.26E-13	1.36E-10
LCN2	7.0	5.47E-13	9.73E-11	8.0	5.43E-09	6.28E-06	12.8	2.17E-23	2.51E-20
LGALS3	2.1	1.06E-14	2.73E-12	1.9	2.91E-02	7.55E-01	2.4	6.25E-11	7.28E-09
LTF	8.1	1.15E-10	1.46E-08	16.0	3.28E-11	7.59E-08	24.0	5.91E-23	5.86E-20
MAOA	5.3	3.64E-34	1.68E-30	4.5	1.00E-05	3.38E-03	5.6	1.39E-19	6.86E-17
MARCH8	2.2	4.18E-12	6.43E-10	2.0	2.08E-01	1.00E+00	2.0	1.40E-06	4.41E-05
MCEMP1	2.2	1.55E-03	2.33E-02	5.5	1.05E-08	9.68E-06	5.1	1.84E-21	1.22E-18
MFSD2B	2.3	2.59E-14	6.31E-12	1.6	1.40E-01	1.00E+00	2.2	6.34E-10	5.26E-08
MKRN1	2.2	1.95E-10	2.31E-08	1.9	2.57E-01	1.00E+00	2.2	5.22E-07	1.89E-05
MMP8	15.5	1.36E-28	3.13E-25	20.9	2.30E-22	1.59E-18	31.0	6.87E-35	4.76E-31
MRC2	2.3	9.64E-11	1.24E-08	1.8	2.07E-01	1.00E+00	2.5	7.73E-07	2.65E-05
MS4A3	2.7	3.18E-10	3.58E-08	5.7	1.48E-10	2.56E-07	2.6	3.67E-06	9.74E-05
MXI1	2.1	1.60E-11	2.29E-09	2.2	8.00E-02	1.00E+00	1.9	1.67E-04	2.32E-03

	RA improved (n=8)			RA worsened (n=3)			Healthy (n=5)		
**Genes**	**FC**	**p-value**	**q-value**	**FC**	**p-value**	**q-value**	**FC**	**p-value**	**q-value**
MYL4	2.8	2.17E-15	6.67E-13	1.8	4.20E-02	8.50E-01	3.2	1.00E-13	2.24E-11
OLFM4	19.1	3.91E-21	4.18E-18	20.2	2.13E-18	7.39E-15	42.8	0.00E+00	0.00E+00
OLR1	2.1	5.78E-07	3.30E-05	2.2	4.07E-04	6.05E-02	1.8	4.88E-05	8.51E-04
OR2W3	2.4	1.23E-17	6.34E-15	2.3	2.90E-02	7.55E-01	3.2	5.16E-15	1.35E-12
OSBP2	2.8	4.16E-17	1.80E-14	2.6	2.37E-02	6.84E-01	3.6	5.42E-13	1.04E-10
PAQR9	2.4	4.32E-08	3.17E-06	1.4	3.21E-01	1.00E+00	2.2	1.25E-04	1.84E-03
PITHD1	3.0	3.53E-18	2.22E-15	2.4	3.65E-02	8.11E-01	2.7	1.39E-08	7.86E-07
PLEK2	2.0	5.60E-18	3.11E-15	1.9	2.75E-02	7.37E-01	2.4	1.34E-09	1.05E-07
PLSCR1	2.1	8.05E-06	3.35E-04	1.3	6.54E-01	1.00E+00	1.2	1.30E-01	3.06E-01
PLSCR2	2.2	1.18E-06	6.02E-05	1.4	4.92E-01	1.00E+00	1.3	1.38E-01	3.18E-01
PLSCR4	2.8	1.91E-17	8.81E-15	1.4	4.10E-01	1.00E+00	2.0	1.23E-05	2.67E-04
PNP	2.0	5.17E-15	1.43E-12	1.9	6.63E-03	3.64E-01	1.7	6.15E-06	1.51E-04
PRDX2	2.2	2.88E-13	5.48E-11	1.7	4.87E-02	8.93E-01	2.9	1.00E-22	8.18E-20
PTX3	2.6	1.77E-07	1.14E-05	3.9	2.57E-06	1.11E-03	2.5	2.16E-07	8.90E-06
RAB3IL1	2.0	8.84E-09	7.81E-07	1.9	1.67E-01	1.00E+00	2.7	2.39E-08	1.25E-06
RAP1GAP	2.5	8.97E-08	6.25E-06	4.5	1.97E-05	5.47E-03	5.3	4.75E-24	5.99E-21
RBM38	2.0	4.58E-09	4.29E-07	1.9	1.78E-01	1.00E+00	2.5	3.28E-09	2.22E-07
RETN	4.7	1.60E-08	1.35E-06	9.5	5.44E-14	1.51E-10	6.1	5.30E-23	5.65E-20
RGCC	2.1	3.83E-15	1.08E-12	1.4	1.11E-01	1.00E+00	1.5	9.41E-03	5.19E-02
RGS10	2.3	3.34E-17	1.50E-14	1.5	1.26E-01	1.00E+00	2.0	2.54E-08	1.32E-06
RHAG	2.1	1.97E-10	2.31E-08	1.7	8.08E-02	1.00E+00	1.8	1.29E-02	6.48E-02
RHCE	2.3	2.86E-12	4.50E-10	2.2	3.29E-03	2.43E-01	1.9	9.23E-06	2.12E-04
RIOK3	2.2	3.38E-14	8.09E-12	2.1	9.53E-02	1.00E+00	1.8	1.05E-04	1.59E-03
RNF14	2.1	8.48E-14	1.81E-11	1.6	1.12E-01	1.00E+00	1.6	1.07E-03	1.01E-02
RPIA	2.1	2.29E-15	6.90E-13	1.8	8.51E-02	1.00E+00	1.5	4.42E-03	2.95E-02
RPL21	2.0	3.87E-07	2.31E-05	1.6	1.40E-01	1.00E+00	2.2	4.22E-11	5.18E-09
RPL36AL	2.1	1.29E-04	3.54E-03	1.6	1.79E-01	1.00E+00	2.3	1.61E-08	8.91E-07
RPL7	2.2	4.98E-08	3.56E-06	1.8	1.74E-01	1.00E+00	2.5	3.37E-13	6.76E-11
RSAD2	2.6	3.77E-03	4.31E-02	0.4	2.13E-01	1.00E+00	0.4	3.13E-03	2.28E-02
S100A12	2.2	2.98E-04	6.81E-03	5.3	3.57E-04	5.69E-02	5.3	1.57E-22	1.21E-19
S100A8	2.2	5.61E-05	1.78E-03	4.9	2.50E-03	2.14E-01	4.9	9.33E-25	1.41E-21
SELENBP1	2.8	4.05E-18	2.34E-15	3.0	2.04E-02	6.41E-01	4.2	4.43E-19	1.98E-16
SELK	2.1	3.65E-13	6.85E-11	1.6	3.66E-02	8.11E-01	2.1	1.04E-10	1.07E-08
SERPINB10	2.3	3.63E-06	1.62E-04	3.1	1.66E-05	4.99E-03	2.8	8.74E-08	4.07E-06
SIAH2	2.3	6.24E-16	2.47E-13	2.4	3.35E-02	7.85E-01	1.9	9.40E-07	3.13E-05
SIGLEC1	3.1	1.55E-05	5.90E-04	0.5	3.41E-01	1.00E+00	0.7	2.74E-01	4.83E-01
SLC14A1	2.5	2.17E-22	3.01E-19	2.0	2.37E-02	6.84E-01	2.1	1.08E-06	3.53E-05
SLC1A5	2.1	2.05E-15	6.45E-13	2.1	2.09E-02	6.47E-01	2.1	1.87E-09	1.40E-07
SLC45A3	0.4	8.12E-08	5.69E-06	0.4	2.06E-03	1.94E-01	0.3	3.20E-11	4.15E-09
SLC4A1	2.5	4.25E-14	9.99E-12	2.9	6.21E-02	9.47E-01	3.8	3.73E-15	1.02E-12
SLC6A8	2.0	5.44E-14	1.24E-11	1.8	1.24E-01	1.00E+00	2.3	2.28E-06	6.47E-05
SLFN14	2.5	1.09E-15	3.79E-13	2.0	5.13E-03	3.16E-01	1.9	9.11E-07	3.07E-05
SLPI	2.4	2.39E-04	5.69E-03	2.2	7.51E-02	1.00E+00	4.3	1.83E-27	3.17E-24
SNCA	2.3	1.79E-10	2.18E-08	1.6	3.62E-01	1.00E+00	2.3	1.78E-05	3.69E-04
SOX6	2.0	1.28E-13	2.69E-11	1.6	1.35E-01	1.00E+00	1.8	2.10E-05	4.19E-04
SPTA1	2.7	6.95E-16	2.53E-13	2.4	1.35E-03	1.53E-01	2.8	1.43E-13	3.05E-11
SPTB	2.2	2.19E-10	2.55E-08	3.0	4.62E-03	2.98E-01	3.6	2.42E-20	1.34E-17
SRRD	2.1	1.04E-17	5.53E-15	1.5	1.18E-01	1.00E+00	1.7	5.48E-05	9.31E-04
STOM	2.3	3.27E-15	9.45E-13	1.8	9.82E-02	1.00E+00	1.8	2.79E-07	1.12E-05

	RA improved (n=8)			RA worsened (n=3)			Healthy (n=5)		
**Genes**	**FC**	**p-value**	**q-value**	**FC**	**p-value**	**q-value**	**FC**	**p-value**	**q-value**
STRADB	2.2	5.07E-12	7.65E-10	1.8	2.52E-01	1.00E+00	1.9	1.25E-03	1.13E-02
TAS2R40	2.6	3.14E-06	1.44E-04	4.4	7.33E-07	4.07E-04	5.2	5.19E-21	3.13E-18
TCN1	4.1	8.07E-16	2.87E-13	5.8	3.91E-05	9.69E-03	4.4	7.41E-20	3.81E-17
TIGD3	0.4	6.23E-14	1.39E-11	0.9	5.04E-01	1.00E+00	0.9	5.50E-01	7.28E-01
TLR5	2.0	1.43E-04	3.84E-03	2.6	1.14E-02	4.84E-01	2.3	9.28E-11	9.82E-09
TMCC2	2.7	3.96E-18	2.34E-15	2.9	3.27E-03	2.43E-01	3.1	1.27E-12	2.31E-10
TMOD1	2.7	5.47E-25	8.43E-22	2.4	2.44E-02	6.88E-01	2.6	4.75E-15	1.27E-12
TNS1	2.1	7.00E-11	9.33E-09	2.1	1.50E-01	1.00E+00	2.6	2.99E-09	2.09E-07
TOP2A	2.0	7.03E-07	3.85E-05	2.5	8.81E-05	1.85E-02	1.1	7.88E-01	8.88E-01
TRAK2	2.1	2.44E-13	4.90E-11	1.7	1.40E-01	1.00E+00	1.8	3.16E-05	5.91E-04
TRIM10	2.7	5.44E-20	4.44E-17	2.5	6.23E-03	3.57E-01	3.1	1.40E-15	4.04E-13
TRIM58	2.4	4.48E-17	1.83E-14	2.7	5.52E-02	9.29E-01	2.7	1.97E-10	1.92E-08
TSPAN5	2.0	8.06E-13	1.40E-10	1.7	2.01E-01	1.00E+00	1.7	1.07E-03	1.01E-02
UBB	2.6	1.07E-08	9.13E-07	1.6	3.43E-01	1.00E+00	4.9	3.81E-11	4.74E-09
UBE2O	2.1	1.71E-13	3.49E-11	1.9	4.43E-02	8.60E-01	2.5	1.02E-18	4.30E-16
XK	3.3	6.46E-27	1.28E-23	2.6	1.45E-02	5.45E-01	2.6	1.53E-07	6.67E-06
YOD1	2.0	1.71E-10	2.10E-08	1.8	2.63E-01	1.00E+00	1.7	4.27E-03	2.89E-02
