# Supplementary figures and images for: Pregnancy-induced gene expression changes in vivo among women with rheumatoid arthritis: a pilot study
Source: Arthritis Res Ther. 2017 May 25;19:104. doi: 10.1186/s13075-017-1312-2 (PMC5445464; doi:10.1186/s13075-017-1312-2)

**Figure S1**

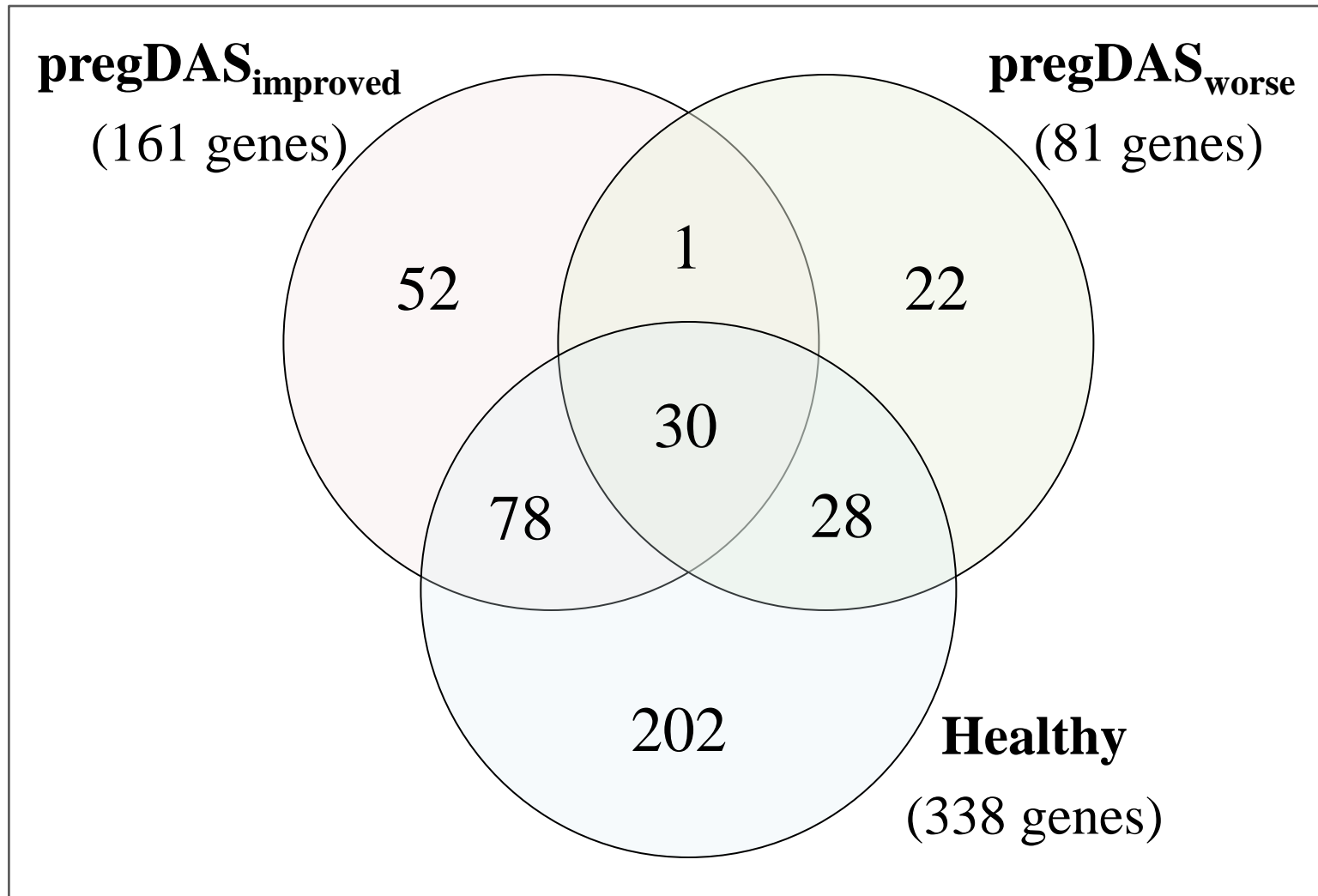

Supplement: Supplementary file 2 — Numbers of genes differentially expressed (q<0.05, FC≥2) in the three groups of women. (Note: These numbers are provided only to give context, given the small sample sizes). (PDF 5 kb) [file 13075_2017_1312_MOESM2_ESM.pdf]
